# The Polyphenol Profile and Antioxidant Potential of Irradiated Rye Grains

**DOI:** 10.1155/2021/8870754

**Published:** 2021-01-16

**Authors:** Dorota Gumul, Wiktor Berski

**Affiliations:** Department of Carbohydrates Technology, University of Agriculture in Krakow, Balicka 122, 30-149 Kraków, Poland

## Abstract

The irradiation process extends the cereal grain storage period, but also affects their chemical composition and antioxidants properties. The aim of this study was to analyze the effect of gamma irradiation on the content of total polyphenols, flavonoids, and tannins as well as the quantitative and qualitative profile of polyphenols in rye grains. The potential antioxidant capacity was also evaluated. The irradiation process resulted in an average increase of 10% of the total phenolic content as compared to the raw material, with each of the analyzed varieties reacting in different manners. The amount of tannins increased after irradiation at a constant level regardless of the applied gamma ray doses in the all analyzed rye grain varieties. The antiradical and antioxidant activity of rye grains after the irradiation process did not change or was reduced.

## 1. Introduction

Cereal grains are excellent source of health-promoting compounds like dietary fiber, vitamins, mineral components, and also polyphenol antioxidants [1], which are mainly concentrated in the grain outer layer. Therefore, cereal consumption is recommended as the preventive measure for chronic diseases such as obesity, coronary heart diseases (CHD), diabetes, and some types of cancer [2].

Among cereals, rye deserves special attention, due to its specific chemical composition, i.e., higher contents of soluble dietary fiber (SDF) including pentosans, elements (Ca, Fe, I, F), lysine, oleic acid, vitamin E, and also with a wide range of polyphenols including phenolic acids (PA) as compared to commonly consumed wheat [3, 4]. It can be therefore said that rye fully deserves the name of health-supporting grain.

The content of polyphenols and their activity can depend on many factors, including variety and degree of maturity and also can be changed as a result of technological processing [5]. It should be noted that the level of antioxidants in rye grain can be changed due to different treatments applied before storage, like for example, the irradiation process. Irradiation reduces the total number of microorganisms, destroys insects, extends the shelf life, and reducing the amount of antinutritional factors [6]. Therefore, it is essential to evaluate the effect of irradiation treatment on the amount and composition of polyphenols and their activity in rye grain. Research to date has focused on changes in the content of nutrients in cereal grains subjected to irradiation, and the only exception was rice, where changes in phenolic compounds were investigated [2].

The aim of this study was to analyze the influence of the applied gamma irradiation at two doses (3 kGy and 10 kGy) on the content of total polyphenols, flavonoids, and tannins, as well as the quantitative and qualitative profile of polyphenols in rye grains (Amilo, Rostockie, and Agrikolo varieties). The effect of such treatments on the potential antioxidant capacity of these raw materials was also investigated.

## 2. Materials and Methods

### 2.1. Materials

The research material consisted of grains of three rye varieties: Amilo (ZA), Rostockie (ZR), and Agrikolo (ZEA) from the Danko-Laski Plant Breeding Station (Poland). They were used as a raw material (control). Rye grains were subjected to gamma radiation treatment at two irradiation doses: 3 kGy (ZA-3 kGy, ZR-3 kGy and ZEA-3 kGy) and 10 kGy (ZA-10 kGy, ZR-10 kGy and ZEA-10 kGy).

The irradiation process was carried out in duplicate. Radiation source was Co-60 installed in panoramic Ob-Servo-D equipment (Hungary), and its activity was 2200 TBq (60KCi). The sample size was 2 kg. The two doses used were 3 and 10 kGy. After irradiation treatment, the rye samples were stored at room temperature in dark for six months. Samples were then milled and passed through a 100 mesh sieve on a Cyclotec 1093 sample mill (Foss Tecator). Rye flours were sealed in air-tight plastic bags and stored at room temperature in dark until use in about two weeks.

### 2.2. Methods

The following analyses were performed:

The quantitative and qualitative composition of phenolic acids (PA) from the hydroxycinnamic group was determined by high-performance liquid chromatography (HPLC) using a Merck-Hitachi L-7455 chromatograph with diode detector. The detector cooperated with the L-7100 pump and the D-7000 HSM Multisolwent Delivery System reagent mixing system. The separation was carried out on a LiChroCART® 125-3 Purospher® RP-18 (5 *μ*m) Merck column, which was thermostated at 30°C. An 80% solution of acetonitrile in 4.5% formic acid (reagent A) and 2.5% acetic acid (reagent B) was used as eluent, at a flow of 1 cm^3^/min, according to the gradient: the concentration of reagent A was increased linearly up to 7 min from 0% to 15%, then up to 15 min to 20% and 16 min to 100% after 10 min column elution; again, the concentration of solution A was lowered to 0% to stabilize the column for 10 min until the next sample injection. During the analysis, the solutions were degassed in a Merck device. Analysis was carried out at wavelength *λ* =320 nm with respect to PA (caffeic, sinapic, ferulic, p-coumaric). The compounds were identified by spectra in the range from 200 nm to 600 nm and retention times compared to standards.

The antioxidant compound content (total phenolic compounds—TPC, flavonoids, and tannins) and antiradical and antioxidant activities were determined in the ethanol extracts. 0.6 g of the sample was dissolved in 30 cm^3^ 80% ethanol, shaken in a darkness for 120 minutes (electric shaker: type WB22, Memmert, Schwabach, Germany), and centrifuged (15 min., 4500 rpm.) in centrifuge (type MPW-350, MPW MED. Instruments, Warsaw, Poland). The supernatant was decanted and stored at -20°C for further analyses for a period of one week.

Determination of the total polyphenol content (TPC) was done by spectrophotometric methods using Folin-Ciocalteu reagent [7]. 5 cm^3^ of ethanol extract was diluted to 50 cm^3^ using distilled water. Next, 5 cm^3^ of previously diluted extract was taken, and 0.25 cm^3^ Folin-Ciocalteau reagent was added (previously diluted with distilled water 1 : 1 v/v) and 0.5 cm^3^ of 7% Na_2_CO_3_. Then, it was vortexed (WF2 type, Janke & Kunkel, Staufen, Germany) and was stored for 30 min. in darkness. Then, absorbance at *λ* = 760 nm was measured using Helios Gamma, 100–240 spectrometer (Runcorn, England). Results were calculated and expressed as catechin mg/g dm.

The content of flavonoids was evaluated using a spectrophotometrical method [8]. 0.5cm^3^ of ethanol extract was vortexed (WF2 type, Janke & Kunkel, Staufen, Germany) with 1.8cm^3^ of distilled water and 0.2cm^3^ of 2-aminoethyl-diphenylborinate reagent. Then, absorbance was measured at *λ* = 404 nm using Helios Gamma, 100–240 spectrometer (Runcorn, England). The flavonoid content was expressed rutin mg/g dm.

Content of condensed tannins (proanthocyanidins) was determined by the spectrophotometric method [9]. 3cm^3^ of vanillin in methanol and 1.5 cm^3^ of concentrated HCl and 0.05cm^3^ of ethanol extract were vortexed (WF2 type, Janke & Kunkel, Staufen, Germany), and then absorbance at *λ* = 500 nm was measured using spectrophotometer (Helios Gamma, 100–240, Runcorn, England). The amount of tannins was expressed as catechin mg/g dm.

Antiradical activities were assessed by ABTS (2,2′-azino-bis(3-ethylobenzothiazoline-6-sulphonic acid)-diamonium salt) [10] and with 2,2-diphenyl-1-picrylhydrazyl (DPPH) [11] methods. The antioxidant activity was assessed using the FRAP (Ferric Reducing Ability of Plasma) method [12].

Briefly, the antiradical activity by ABTS was measured on ethanol extract vortexed with ABTS (WF2 type, Janke & Kunkel, Staufen, Germany), and absorbance was measured at *λ* = 734 nm spectrophotometer (Helios gamma 100-240, Runcorn, England). Next, the second reading was made after six minutes at the same wavelength. The antiradical activity by DPPH was measured in ethanol extracts (1cm^3^) mixed with 4cm^3^ of DPPH solution (0.012 g DPPH diluted with ethanol up to 100cm^3^). Absorbance was measured at *λ* = 515 nm. Antiradical activities were given as trolox equivalent antioxidant capacity (TEAC).

The antioxidant activity by the FRAP method was measured as follows. To 10 cm^3^ test tubes, 3.3 cm^3^ of acetate buffer (pH = 3.6), 0.330 cm^3^ of FeCl_3_ (20 mmol/dm^3^), and 0.330 cm^3^ of tripyridyltriazine (10 mmol/dm^3^ in 40 mmol/dm^3^ HCl) were added and heated in a water bath at a temperature of 37°C for 5 minutes. Then, 0.330 cm^3^ of the ethanol extracts of the analyzed material was added. Absorbance at *λ* = 593 nm was measured after 15 min in disposable plastic absorption cells using a spectrophotometer UV-530 (Jasco, Japan). The antioxidant activity was given in mMFe/kg dm.

### 2.3. Statistical Analysis

All measurements were performed in at least in duplicate, and the obtained results were subjected to the analysis of variance (ANOVA) using the Statistica 13 statistical software package. The significance of differences between the average values was verified by Duncan's test at *α* ≤ 0.05.

## 3. Results and Discussion

### 3.1. Effect of Gamma Irradiation on Qualitative and Quantitative Polyphenol Profile in Rye

Research on the individual polyphenols was focused on the content of six PA and apigenin in rye grains before and after the irradiation process. Five of them are classified as cinnamic acid derivatives (sinapic, ferulic, diferulic, caffeic, and p-coumaric), and on other hand, vanilic is a derivative of benzoic acid. Contents of the abovementioned phenols are shown in Table 1.

The content of individual PA and apigenin in the three rye varieties was in the following order: Agrikolo (ZEA) (47.04 mg/100 g dm) > Rostockie (ZR) (42.02 mg/100 g dm) > Amilo (ZA) (38.70 mg/100gs dm) (Table 1). In accordance with previous studies [1, 13], it was shown that ferulic, sinapic, caffeic, p-coumaric, and vanilic acids were the main polyphenol antioxidants in rye grains, with their amount depending mostly on the variety.

Total amount of identified phenolic compounds increased on average by 20% in grains of two varieties (ZA and ZR) after the irradiation process, with the exception for ZEA variety (Table 1). It was noticed that in irradiated ZA grains, the content of individual PA (sinapic, ferulic, and vanilic) and apigenin increased in the range of 20-67% and remained stable regardless of the applied irradiation dose as compared to the raw material (control). The exceptions in this respect were diferulic, caffeic and p-coumaric acids, and the level of which before and after irradiation treatment was constant (Table 1). In the case of ZR grains, it was noted that when a lower irradiation dose was applied (3 kGy), the content of PA: sinapic, diferulic, caffeic, and apigenin did not change, and the amount of other PA increased in the grains irradiated with this dose, as compared to the raw material (control). In the case of ferulic, p-coumaric, and vanilic, this increase was 17.0, 14.5, and 27.6% in relation to not treated material. It was also observed that in ZR grains, the content of PA (sinapic, ferulic, diferulic, caffeic, and p-coumaric) was higher when a higher irradiation dose was applied, but in contrast to vanilic acid, which amounts decreased (Table 1). ZEA grains treated with 10 kGy dose were characterized by a similar content of sinapic, diferulic, p-coumaric, and ferulic acids, as well as a reduced content of caffeic acid and apigenin when compared to the raw material—control (Table 1). In the case of the 3 kGy dose, a 12% increase in the content of sinapic acid was noted, and the amount of remaining PA (ferulic, diferulic, caffeic and p-coumaric, and vanilic acids) did not change in relation to the raw material, while the amount of apigenin decreased by 35% in relation to raw material—control (Table 1).

Phenolic compounds present in cereal grains like PA and apigenin are biologically active ingredients and constitute an integral part of cell walls composed mainly of fiber. Free PA is found in cereal grains in small amounts. They are most often present in bound form, in the form of esters and glycosides, as elements of complex structures of lignins and tannins. Phenolic compounds can bound with other food ingredients, i.e., proteins, carbohydrates, and fatty acids [14, 15]. According to Shao et al. [6], gamma irradiation treatment displayed much stronger effects on the bound phenols than on the free ones. Ferulic acid, which is the dominant PA in cereals, most commonly is occurring with arabinoxylans connected by covalent bonds. Gamma irradiation can partially break down these bonds, making this acid more susceptible to extraction (e.g., HPLC sample preparation), resulting in increased content in plant material (Table 1). Similar situation was observed for other PA, the increase of which was significant in rye grains after the irradiation process (Table 1).

The irradiation process releases, among others, ferulic acid, which increases its bioavailability [15]. Therefore, it has a greater impact as a potential antioxidant in the human diet, which is extremely valuable especially in the context of the chemopreventive role of this ingredient. Ferulic acid is considered to be an anticancer agent. This works proves that the rye grain irradiation process not only reduces the total number of microorganisms, destroys insects, extends the shelf life, as well as reducing the amount of antinutritional factors, and also can contribute to the increase of some important, from nutritional point of view, polyphenol content. It can be observed that in rye grains after gamma ray irradiation treatment, ferulic acid content increased by 24% as compared to not treated one (control). So, it can be applied as health promoting matrix in dietetic food or pharmaceutical production. As was previously mentioned, gamma irradiation releases endogenic ferulic acid from arabinoxylans, and for that reason, it becomes more available. It can be suggested that such treatment can be applied in production of novel cereal-based food supplements.

However, in the studies related to the effect of gamma irradiated (2 to 10 kGy) rice samples (black, red and white) [2], it was found that these samples reacted differently to the same irradiation dose.

In the case of black rice, a dose of 2-8 kGy contributed to the reduction of the PA content (from 15 to 30%), while the 10 kGy dose essentially did not change their amount. For red and white rice, the effect of applied doses of gamma irradiation from 2 to 10 kGy resulted in the reduced amount of PA. Also, presented results proved that rye varieties differently responded to the same irradiation doses (3 and 10 kGy), which was confirmed by our results (Table 1). According to Zhu et al. [2], it is possible that gamma irradiation can disrupt the PA (especially free phenols), and as a result, reducing their amount. But on the other hand, it can activate some enzyme inducing PA synthesis. The balance between the synthesis and breakdown of PA depends on the applied irradiation dose during cereal processing. It can therefore be suggested that the effect of irradiation on cereal grains depends on many factors.

### 3.2. Effect of Gamma Irradiation on Polyphenols, Flavonoids, and Tannin Content in Different Rye Varieties

The total polyphenol content (TPC), flavonoid, and tannin content in rye before and after irradiation (3 and 10 kGy) are given in Table 2.

TPC calculated as catechin in grains of rye varieties: ZA, ZR, and ZEA were, respectively, 1.86, 2.14, and 2.11 mg catechin/g dm (Table 2). These values are difficult to compare with other literature data, due to the different of extraction and determination methods applied and also results expressing methods applied by other authors [16]. Zieliński and Troszyńska [17] using the buffer extraction method (PBS) obtained TPC of 0.94 mg catechin/g in rye grains, and applying 80% methanol-0.65 mg catechin/g rye, while Zieliński et al. [18] determined TPC in rye grains at level 1.4 mg catechin/g rye, extracting plant material in phosphate buffer. In this work, ethanol extraction was applied, and also other extraction conditions were used; therefore, the results obtained were higher than those obtained by Zieliński and Troszyńska [17] and Zieliński et al. [18].

The irradiation process of rye grains caused an average increase of TPC about 10% in relation to the raw material (control), with each of the investigated varieties reacting in different manners to the same doses of gamma irradiation (Table 2). In the case of irradiated ZA grains, the overall content of polyphenols did not change for 3 kGy dose, but for 10 kGy increased by 11%, as compared to raw material (control). Preservation by irradiation of ZR grains resulted in an increase in TPC, higher at 10 kGy (17% increase relative to raw material) than for 3 kGy dose (9% increase relative to raw material). For ZEA grains, the higher total amount of polyphenols was observed at 3 kGy dose (15% increase relative to raw material) than for 10 kGy (6% increase in comparison to raw material) (Table 2). An increase in TPC in irradiated orange peels with an increase in dose from 1 to 2 kGy was observed by Moussaid et al. [19]. Similarly, Harrison and Were [20] found an increase in polyphenols by 45% in almond hulls subjected to irradiation (4 kGy) and by 20% at a dose above 12 kGy. These authors attributed this to the release of polyphenols from glycosidic linkages and the breakdown of high-molecular compounds with the release of low-molecular polyphenols. However, in the studies of Shao et al. [6], it was reported that lower dose of irradiation treatment decreased the free phenolic content of white rice, but a higher dose of gamma rays (above 4 kGy) increased the content of these compounds, while the amount of bound of the phenolic content increases (at doses from 4 to 10 kGy), which consequently increases the TPC in this plant material as well as the other two analyzed raw materials (red and black rice). Other authors [21] claimed that TPC in soybean seeds decreased with increasing dose of gamma rays. The discrepancies described above in the results of studies conducted by various authors regarding the impact of gamma rays on the content of phenolic compounds can be explained by the different compositions of this group in different types of raw materials. And on the other hand, the already mentioned balance between the destructive action of gamma rays on bonds which results in molecular changes and the activation of some enzyme inducing the synthesis of phenolic compounds.

In the case of two polyphenols subgroups: flavonoids and tannins, it was found that each of the analyzed rye varieties reacted differently to the same doses of applied gamma rays in the respect of flavonoids. The amount of tannins increased after irradiation at a constant level regardless of the dose of gamma rays in the all analyzed rye varieties (Table 2). For grains of ZA variety after the application of 3 kGy dose, stabilization in the flavonoid level was observed, and for 10 kGy dose, 45% increase in the flavonoids amount in relation to the raw material (control) was noted. After irradiation, the flavonoids in ZR grains increased irrespective of the applied dose by about 25% in relation to the raw material, whereas for ZEA grains, no change was observed.

In the case of tannins, their content increased after the irradiation process irrespective of the rye variety and irradiation dose (3 and 10 kGy) in the range from 27 to 70% in relation to the not treated grains (control) (Table 2). According to Costa de Camargo et al. [22], the effect of gamma irradiation on procyanidin in the peanut skin resulted in their depolymerization or in conversion of B type dimers into type A, which means that their quantity according to the above mentioned authors can increase in plant material after irradiation, which was also observed in the results of this work (Table 2).

### 3.3. Effect of Gamma Irradiation on Antioxidative Potential of Different Rye Varieties

The antiradical activity of rye grains before and after irradiation was determined using two free synthetic radicals: DPPH and ABTS (radical cation) are presented in Table 3. Of the all three analyzed rye varieties, the highest antiradical activity was observed for ZEA variety grains (6.07 mMTx/kg dm–DPPH, 17.86 mMTx/kg dm-ABTS), the middle for ZR variety (5.78 mMTx/kg dm–DPPH, 16.81mMTx/kg dm ABTS), and the lowest for ZA variety (5.7mMTx/kg dm DPPH, 14.21 mMTx/kg dm ABTS) (Table 3). The antioxidant (and antiradical) activity depends, among others, not only on the total amount of phenolic compounds but also on their individual composition in plant material [23]. Above mentioned authors determined the highest free radical scavenging capacity of DPPH for caffeic acid, then by sinapic and ferulic acids. The highest rate of DPPH reduction by ZEA extracts should be probably explained by the highest total content of identified polyphenols (Table 1), as well as by higher share of acids with high efficiency of DPPH free radical scavenging (sinapic and ferulic acid) in this rye variety, as compared to other varieties (Table 1).

The antiradical activity of rye grains after irradiation has been reduced or has not changed as compared to the not treated grains (control), both for DPPH and ABTS methods (Table 3). The decrease in the antiradical activity in irradiated grains was not related to TPC, as their quantity increased after irradiation (Tables 2 and 3). It can therefore be assumed that the composition of polyphenols present in this plant material influences the antiradical activity (Table 1). Karamać et al. [23] found the highest scavenging capacity of DPPH that had caffeic, then sinapic and ferulic acid. ZA irradiated grains (by both doses) were characterized by almost identical antiradical activity: 5.74 mMTx/kg dm DPPH, 14.30, and 15.21 mMTx/kg dm ABTS quite similar as in raw material (Table 3), which can be explained by the constant content of individual PA and especially the most effective caffeic acid (Tables 1 and 3). Irradiated ZR variety grains, irrespective of the irradiation dose, showed reduced antiradical activity, as compared to the raw material (DPPH method), and the similar capacity (ABTS) at the 3 kGy dose and reduced by 6% at the 10 kGy dose, in relation to control. In the case of irradiated ZEA grains, a greater decrease in the antiradical activity in relation to the raw material was noted at a lower irradiation dose, determined by both methods (Table 3).

The low antiradical activity of irradiated ZR and ZEA grains did not result from the composition of the individual phenolic compounds or TPC, but could be due to a number of other factors. It should be also taken into consideration that gamma rays cause, among others, changes in molecular conformation, disruption of covalent bonds, and formation of free radicals and oxidative changes [24–26]. In addition to polyphenols, other endogenous antioxidant components, e.g., phytates, as well as other antioxidative and pro-oxidative compounds formed during the irradiation process, are involved in antiradical and antioxidative activity. Low antiradical activity of cereal grains after irradiation or stabilization of the antioxidant potential in relation to nonirradiated grains was most probably related to the fact that irradiation generated pro-oxidizing compounds, i.e., reactive water radiolysis products, which contribute to the reduced antiradical potential of the product thus obtained. Lampart-Szczapa et al. [27] noticed a similar decrease in the antiradical properties of irradiated lupine flour. On the other hand, this stabilization could be due to the effect of phytic acid, which according to Ahn et al. [28] irradiated with 10 and 20 kGy doses showed significantly greater antiradical and antioxidant activity than before irradiation. In cereals, phytic acid is present in quite significant amounts [4] and therefore, it could create the antiradical activity of cereal grains.

Also, the antioxidant activity determined by the FRAP method in rye grains before and after irradiation did not change or decreased after this treatment as for ZEA variety by 10%, in comparison to not irradiated grains (Table 3). As in the case of the antiradical activity of this material, it was most likely the result of the formation of pro-oxidative forms, which became the reason for the decrease in the antioxidant activity [27] and the increase in the antioxidant activity of phytic acid after irradiation, which consequently balanced the losses of this activity and followed by the stabilization of this antioxidant activity in grains of ZA and ZR varieties before and after the irradiation process (Table 3).

In the research of de Camargo et al. [22] regarding the effect of gamma irradiation on the antioxidant activity of the peanut skin, it was observed a slight decrease or stabilization of the antiradical activity determined by DPPH and ABTS, and the change in this activity largely depended on doses of gamma rays (2 or 5 kGy).

## 4. Conclusions

1. The irradiation process resulted in an increase of the phenolic acid content, dominating in rye grain, i.e., sinapic, ferulic p-coumaric, and vanilic. This increase depended on the applied gamma ray doses and rye variety.
2. The irradiation of rye grain caused an average 10% increase in the total phenolic content in relation to the raw material (control), with each of the analyzed varieties reacting in different ways to the same doses of gamma irradiation.
3. In the case of two subgroups of polyphenols: flavonoids and tannins, it was found that each of the analyzed rye variety reacted differently to the same doses of gamma rays in the case of flavonoids. The amount of tannins increased after irradiation at a constant level, regardless of the applied gamma ray doses, in the all analyzed rye grain varieties.
4. The antiradical and antioxidant activity of Amilo (ZA) and Rostockie (ZR) varieties grains after the irradiation process did not change as compared to the grains not subjected to this operation (control), or was reduced, as for the Agrikolo (ZEA) variety grain.

## Figures and Tables

**Table 1:** The content of the selected polyphenols in rye grains before and after irradiation [mg/100 g dm].

Sample	Sinapic acid	Ferulic acid	Di ferulic acid	Caffeic acid	p-Cumaric acid	Vanilic acid	Apigenin	Total
ZA	5.91 ± 0.22^a^^∗^	25.01 ± 1.94^a^	0.77 ± 0.36^a^	2.79 ± 0.16^a^	1.35 ± 0.29^a^	1.78 ± 0.20^a^	1.06 ± 0.03^a^	38.70
ZA-3 kGy	7.83 ± 0.02^b^	29.27 ± 0.36^b^	0.93 ± 0.02^a^	2.97 ± 0.11^a^	1.51 ± 0.04^a^	2.88 ± 0.06^b^	1.48 ± 0.06^b^	46.88
ZA-10 kGy	7.91 ± 0.06^b^	30.25 ± 0.01^b^	0.89 ± 0.05^a^	2.95 ± 0.11^a^	1.71 ± 0.07^a^	2.99 ± 0.06^b^	1.43 ± 0.02^b^	48.13
ZR	6.41 ± 0.06^a^	25.34 ± 0.10^a^	0.94 ± 0.07^a^	2.61 ± 0.08^a^	1.93 ± 0.14^ab^	2.97 ± 0.06^b^	1.82 ± 0.09^a^	42.02
ZR-3 kGy	6.56 ± 0.04^a^	29.57 ± 0.91^b^	0.86 ± 0.01^a^	2.66 ± 0.22^a^	2.21 ± 0.17^b^	3.79 ± 0.35^c^	1.75 ± 0.29^a^	47.40
ZR-10 kGy	7.71 ± 0.04^b^	35.44 ± 0.81^c^	1.42 ± 0.24^b^	3.75 ± 0.00^b^	2.74 ± 0.29^c^	1.53 ± 0.10^a^	1.79 ± 0.48^a^	54.37
ZEA	7.02 ± 0.36^a^	28.43 ± 3.59^a^	0.89 ± 0.15^a^	2.80 ± 0.47^b^	1.79 ± 0.10^a^	3.53 ± 0.18^a^	2.59 ± 0.15^c^	47.04
ZEA-3 kGy	7.84 ± 0.05^b^	30.35 ± 0.10^a^	0.89 ± 0.05^a^	2.61 ± 0.11^b^	1.83 ± 0.09^a^	3.80 ± 0.04^ab^	1.70 ± 0.10^b^	49.00
ZEA-10 kGy	7.08 ± 0.06^a^	27.97 ± 0.06^a^	0.96 ± 0.11^a^	2.18 ± 0.05^a^	2.08 ± 0.15^a^	4.11 ± 0.11^b^	1.18 ± 0.07^a^	45.60

^∗^Average ± SD; values within variety (section within the column) denoted with the same superscript are not statistically different according to the Duncan test (*α* = 0.05).

**Table 2:** Polyphenols, flavonoids, and total tannin content in grains of rye varieties before and after irradiation.

Sample	Total phenolic content (TPC) [mg catechin/g dm]	Content of flavonoids [mg rutin/g dm]	Content of tannins [mg catechin/g dm]
ZA	1.86 ± 0.03^a^	0.265 ± 0.005^a^	0.325 ± 0.000^a^
ZA-3 kGy	1.77 ± 0.05^a^	0.272 ± 0.013^a^	0.489 ± 0.001^b^
ZA-10 kGy	2.07 ± 0.10^b^	0.385 ± 0.009^b^	0.487 ± 0.002^b^
ZR	2.14 ± 0.00^a^	0.210 ± 0.005^a^	0.338 ± 0.000^a^
ZR-3 kGy	2.34 ± 0.10^b^	0.269 ± 0.020^b^	0.578 ± 0.011^b^
ZR-10 kGy	2.51 ± 0.11^c^	0.257 ± 0.014^b^	0.579 ± 0.000^b^
ZEA	2.11 ± 0.00^a^	0.246 ± 0.012^a^	0.384 ± 0.048^a^
ZEA-3 kGy	2.42 ± 0.05^c^	0.240 ± 0.006^a^	0.490 ± 0.000^b^
ZEA-10 kGy	2.25 ± 0.08^b^	0.253 ± 0.008^a^	0.478 ± 0.000^b^

^∗^Average ± SD; values within variety (section within the column) denoted with the same superscript are not statistically different according to the Duncan test (*α* = 0.05).

**Table 3:** Antiradical activity (measured by DPPH and ABTS methods) and antioxidative properties of rye varieties grain before and after irradiation.

Sample	FRAP[mMFe/kg dm]	DPPH		ABTS	
		TEAC^∗^ [mgTx/g dm]	TEAC [mMTx/kg dm]	TEAC [mgTx/g dm]	TEAC [mMTx/kg dm]
ZA	10.90 ± 0.80^a^^∗∗^	1.42	5.70 ± 0.11^a^	3.56	14.21 ± 0.39^a^
ZA3 kGy	9.89 ± 1.69^a^	1.43	5.74 ± 0.08^a^	3.58	14.30 ± 0.59^a^
ZA10 kGy	9.69 ± 0.88^a^	1.43	5.74 ± 0.08^a^	3.81	15.21 ± 1.20^a^
ZR	11.71 ± 0.01^a^	1.44	5.78 ± 0.06^b^	4.21	16.81 ± 0.74^ab^
ZR3 kGy	11.57 ± 1.71^a^	1.28	5.13 ± 0.04^a^	4.20	16.77 ± 0.62^ab^
ZR10 kGy	11.08 ± 0.68^a^	1.33	5.31 ± 0.21^ab^	3.96	15.80 ± 0.68^a^
ZEA	11.98 ± 0.01^c^	1.52	6.07 ± 0.14^b^	4.47	17.86 ± 0.60^b^
ZEA 3 kGy	11.10 ± 0.43^b^	1.38	5.54 ± 0.06^a^	3.93	15.68 ± 0.81^a^
ZEA 10 kGy	10.49 ± 0.44^a^	1.49	5.96 ± 0.17^b^	4.22	16.87 ± 0.32^ab^

^∗^TEAC: trolox equivalent antioxidant capacity, ^∗∗^average ± SD; values within variety (section within the column) denoted with the same superscript are not statistically different according to the Duncan test (*α* = 0.05).

## Data Availability

The data used to support the findings of this study are included within the article.
